# Low-grade mucinous neoplasms (LAMN) of the appendix in Germany between 2011 and 2018: a nationwide analysis based on data provided by the German Center for Cancer Registry Data (ZfKD) at the Robert Koch Institute (RKI)

**DOI:** 10.1007/s00423-022-02639-w

**Published:** 2022-08-12

**Authors:** Franziska Köhler, Lena Reese, Anne Hendricks, Carolin Kastner, Sophie Müller, Johan F. Lock, Christoph-Thomas Germer, Armin Wiegering

**Affiliations:** 1 Department of General, Visceral Transplantation, Vascular and Pediatric Surgery, University Hospital, University of Wuerzburg, Oberduerrbacherstr. 6, 97080 Würzburg, Germany; 2 Department of Biochemistry and Molecular Biology, University of Wuerzburg, Am Hubland, 97074 Würzburg, Germany; 3 Comprehensive Cancer Center Mainfranken, University of Wuerzburg Medical Centre, Josef-Schneiderstr. 2, 97080 Würzburg, Germany

**Keywords:** LAMN, Low-grade mucinous neoplasm, Appendix, Epidemiology, ZfKD, Germany

## Abstract

**Introduction:**

Low-grade appendiceal mucinous neoplasms (LAMN) are semi-malignant tumors of the appendix which are incidentally found in up to 1% of appendectomy specimen. To this day, no valid descriptive analysis on LAMN is available for the German population.

**Methods:**

Data of LAMN (ICD-10: D37.3) were collected from the population-based cancer registries in Germany, provided by the German Center for Cancer Registry Data (Zentrum für Krebsregisterdaten—ZfKD). Data was anonymized and included gender, age at diagnosis, tumor staging according to the TNM-classification, state of residence, information on the performed therapy, and survival data.

**Results:**

A total of 612 cases were reported to the ZfKD between 2011 and 2018. A total of 63.07% were female and 36.93% were male. Great inhomogeneity in reporting cases was seen in the federal states of Germany including the fact that some federal states did not report any cases at all. Age distribution showed a mean age of 62.03 years (SD 16.15) at diagnosis. However, data on tumor stage was only available in 24.86% of cases (*n* = 152). A total of 49.34% of these patients presented with a T4-stage. Likewise, information regarding performed therapy was available in the minority of patients: 269 patients received surgery, 22 did not and for 312 cases no information was available. Twenty-four patients received chemotherapy, 188 did not, and for 400 cases, no information was available. Overall 5-year survival was estimated at 79.52%. Patients below the age of 55 years at time of diagnosis had a significantly higher 5-year survival rate compared to patients above the age of 55 years (85.77% vs. 73.27%).

**Discussion:**

In this study, we observed an incidence of LAMN in 0.13% of all appendectomy specimen in 2018. It seems likely that not all cases were reported to the ZfKD; therefore, case numbers may be considered underestimated. Age and gender distribution goes in line with international studies with females being predominantly affected. Especially regarding tumor stage and therapy in depth information cannot be provided through the ZfKD-database. This data analysis emphasizes the need for further studies and the need for setting up a specialized registry for this unique tumor entity to develop guidelines for the appropriate treatment and follow-up.

## Introduction

Neoplasms of the appendix are a rare condition and represent merely 0.5% of all neoplasms of the gastro-intestinal system. However, between 0.9 and 1.7% of appendectomy specimens contain a tumor [1, 2]. Approximately 50% of these tumors are described as low-grade appendiceal mucinous neoplasms (LAMN) [3]. LAMN are unique in their histological presentation and form of dissemination. Instead of infiltrative growth as it is usually seen in malignant tumors, they show a pushing margin with low-grade atypia [4]. While LAMN do not metastasize haematogenic or lymphogenic, they can disseminate in the abdominal cavity due to perforation, leading to an accumulation of mucin which is called “pseudomyxoma peritonei” (PMP) [3, 5–8]. LAMN are categorized according to the UICC-Classification (Union for International Cancer Control), focusing on the penetration of mucin into the appendix-wall and the presence of mucin in the abdominal cavity itself. McDonald et al. also established the division into LAMN type I and II [8]. In LAMN type I mucin is confined to the lumen of the appendix. If the tumor progresses to LAMN type II, mucin can be found in the appendiceal wall or outside the appendix [8, 9]. In advanced stages and progression to PMP, mucin is widely distributed into the abdominal cavity [9].

Patients with LAMN often show symptoms of appendicitis with its typical clinical features: pain in the right iliac fossa, fever, nausea, and vomiting along with raised c-reactive protein and leukocytes [4]. In case of progression to PMP, patients additionally present with symptoms of abdominal discomfort and mucinous ascites [8, 10].

In non-perforated early stages (type I) of LAMN, appendectomy seems to be the adequate therapy [7]. In advanced stages (type II), appendectomy, peritonectomy, and additional HIPEC (hyperthermic intraperitoneal chemotherapy) should be considered. In cases of PMP, cytoreductive surgery with additional HIPEC is the advised therapy [7, 8, 11].

Adequately treated patients are reported to have an excellent overall survival and a 5-year recurrence-free survival ranging from 78.3 to 95.1% [7, 12].

Until now, there is no recent valid data about incidence, age, and gender distribution as well as treatment strategies and survival probability of LAMN in Germany. In comparison, Smeenk et al. performed an analysis of the Dutch nationwide pathology database as early as 2008 to investigate the incidence of appendiceal neoplasms and PMP [3].

The German Center for Cancer Registry Data (ZfKD) at the Robert Koch Institute (RKI) in Berlin collects data on cancer patients provided by the regional population-based registries. Therefore, valid information is available on a national level. Cancer confined to the appendix, including LAMN, is not mentioned in the recently revised edition of “Cancers in Germany 2015/2016” by the ZfKD, as it is a very rare condition [13].

The aim of this data analysis is to evaluate incidence, age, and gender distribution as well as the rate of tumor stages, performed therapies and survival probability.

## Methods

Data was provided by the German Center for Cancer Registry Data (ZfKD; https://www.krebsdaten.de/Krebs/DE/Content/ZfKD/zfkd_node.html) which receives data on cancer patients through the local cancer registries of the 16 German federal states. Cancer data is transmitted anonymously and includes gender, month and year of birth, residence, tumor diagnosis according to the TNM-classification, month and year of diagnosis, age at diagnosis, histological findings according to the ICD-O-3 classification, and grading and information about therapy (surgery, radiation, and chemotherapy). Furthermore, data about survival can be evaluated if the observation period is long enough [14].

Formal application for data use and analysis was approved by the ZfKD ethics committee prior to data transmission.

Data on patients reported to the ZfKD between January 2011 and December 2018 with the ICD-10 code *D37.3* (“neoplasm of the appendix of uncertain or unknown behavior”) and ICD-O3 histological code *8480/1* (“low-grade appendiceal mucinous neoplasm”) were analyzed. In 2013, the histological code for LAMN was officially released in the updated version of the ICD-0–3 classification [15, 16].

Data analysis was performed using Microsoft Excel (Version 16.36) and GraphPad Prism (Version 9.0.0 (86)). Graphs and tables were created in GraphPad Prism as well.

**Fig. 1:**
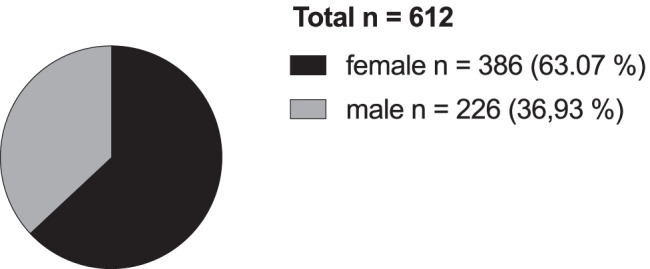
Reported LAMN cases divided by gender

Descriptive analysis was performed if not noted otherwise. Regarding tumor stage and therapy, descriptive analysis was severely limited due to large numbers of missing data (Fig. 1).

Overall survival was analyzed for all patients and furthermore by dividing patients in two subgroups: below and above the age of 55 at date of diagnosis. Statistical significance was calculated using the log-rank-test. *P*-value < 0.05 was defined as significant.

## Results

### Case numbers

Overall, 612 cases of LAMN defined by the ICD-O3 histological code 8480/1 were reported to the ZfKD-database between January 2011 and December 2018. Case numbers were low in 2011 and 2012 and represented 3.92% of the overall reported cases. Throughout the following years, reported cases increased to the tenfold amount from 2015 onward compared to 2011 (Table 1).

**Table 1:** Number, percentage, and division by gender of LAMN cases reported to the ZfKD between 2011 and 2017 in Germany

Year	Number of patients	Male *n* (%)	Female *n* (%)
2011	*n* = 9	*n* = 3 (33.33)	*n* = 6 (66.66)
2012	*n* = 15	*n* = 4 (26.67)	*n* = 11 (73.33)
2013	*n* = 37	*n* = 14 (37.84)	*n* = 23 (62.16)
2014	*n* = 71	*n* = 27 (38.03)	*n* = 44 (61.97)
2015	*n* = 110	*n* = 45 (40.01)	*n* = 65 (59.09)
2016	*n* = 110	*n* = 40 (36.36)	*n* = 70 (63.64)
2017	*n* = 118	*n* = 43 (36.44)	*n* = 75 (63.55)
2018	*n* = 142	*n* = 50 (35.21)	*n* = 92 (64.79)
Overall	***n* = 612**	***n* = 202 (33.01)**	***n* = 410 (66.99)**

Furthermore, a great inhomogeneity in the reporting of cases between the federal states of Germany could be seen. While some states (Hamburg, Hesse, and Baden-Württemberg) did not report any cases during the observation period, others did not report cases in some years or no cases appeared in these years. Results are shown in Table 2. Reasons for the disparity in the reporting process are not available in the dataset.

**Table 2:** Case numbers and percentage divided by federal state between 2011 and 2018. Highlighted in gray are years in which states did not report cases

Federal state	2011	2012	2013	2014	2015	2016	2017	2018	Number (%)
Schleswig–Holstein	2	2	3	4	2	3	3	2	21 (3.43%)
Hamburg	0	0	0	0	0	0	0	0	0 (0%)
Lower Saxony	0	0	1	4	8	15	13	10	51 (8.33%)
Bremen	0	1	2	0	0	4	0	4	11 (1.79%)
North Rhine-Westphalia	4	7	12	30	40	32	45	61	231 (37.75%)
Hesse	0	0	0	0	0	0	0	0	0 (0%)
Rhineland Palatinate	0	0	0	0	1	1	0	5	7 (1.14%)
Baden-Württemberg	0	0	0	0	0	0	0	0	0 (0%)
Bavaria	2	5	8	13	18	24	22	31	122 (19.93%)
Saarland	0	0	0	0	2	1	0	0	3 (0.48%)
Berlin	0	0	3	3	3	3	0	0	12 (1.96%)
Brandenburg	0	0	1	4	2	2	4	0	13 (2.12%)
Mecklenburg Western Pomerania	0	0	1	1	7	1	6	8	24 (3.92%)
Saxony	1	0	5	9	20	12	15	13	75 (12.25%)
Saxony-Anhalt	0	0	0	1	4	8	2	4	19 (3.10%)
Thuringia	0	0	1	2	4	4	8	4	23 (3.76%)

### Gender and age distribution

**Fig. 2:**
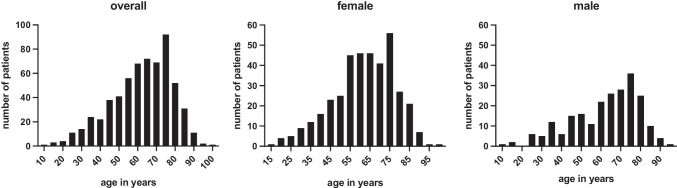
Age distribution of LAMN cases, overall and divided by gender

As shown in Fig. 2 and Table 1, in our study population of 612 patients, 386 patients (63.07%) were female and 226 patients (36.93%) were male (female-male ratio 1.7:1).

Looking at both genders, average patient age was 62.03 years (*SD* = 16.15), ranging from 11 to 97 years. There was no significant difference in mean age of women (61.99 years; *SD* = 15.71) and men (62.09; *SD* = 16.91) (Fig. 2).

### Tumor staging

Tumor staging according to the 7th or 8th TNM-classification was available for the minority of patients; therefore, further analysis on significance was not performed due to scarce data.

**Fig. 3:**
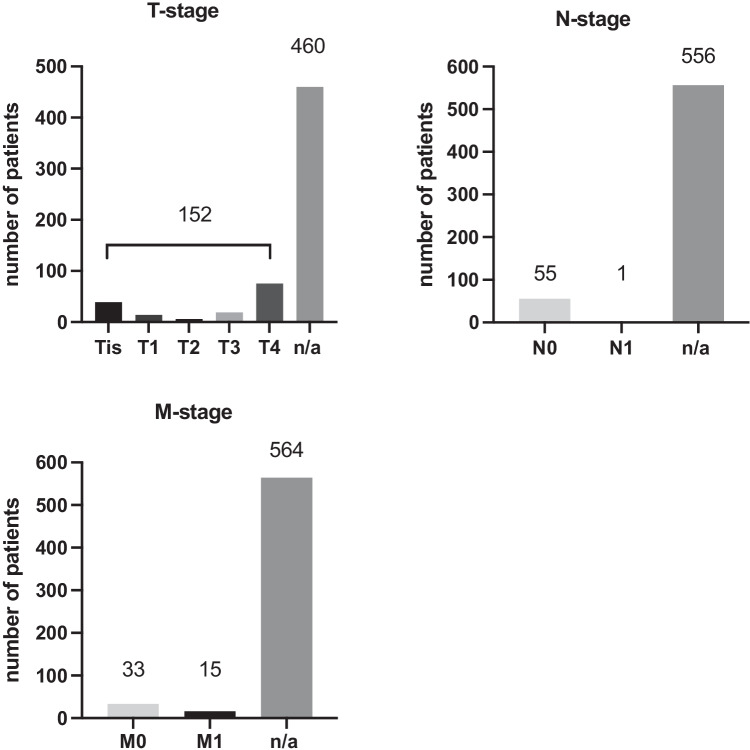
LAMN cases divided by TNM-classification and number of patients with not available TNM-classification; n/a, not available

T-classification was available in 152 cases (24.86%). Of these patients, 49.34% presented with a T4 stage (*n* = 75), 12.5% with T3 (*n* = 19), 3.29% with T2 (*n* = 5), 9.21% with T1 (*n* = 14), and 25.66% with pTis (*n* = 39) as shown in Table 3 and Fig. 3.

**Table 3:** Tumor stages of reported LAMN cases according to the TNM classification, divided by gender

T-stage	Number of patients	Male *n* (%)	Female *n* (%)	Age (years)
pTis	39	11 (28.21)	28 (71.79)	69.56
T1	14	5 (35.71)	9 (64.29)	59.79
T2	5	2 (40)	3 (60)	74.91
T3	19	9 (47.37)	10 (52.63)	61.33
T4	75	*n* = 40 (35.09)	*n* = 74 (64.91)	60.87
Overall	***n* = 152**	***n* = 52 (34.21)**	***n* = 100 (87.72)**	**63.53**

Nodal state was specified in 8.99% (*n* = 56) and was negative (N0) in all of these cases with just one exception (Fig. 3).

The existence of metastases was specified in 48 patients (7.84%), 33 patients did not have metastases (M0), and 15 did present with metastases (M1) (68.75% and 31.25% respectively) (Fig. 3). For nearly all metastatic patients, with only one exception, the tumor stage was available and all of them were reported with a T4-stage. Patients with reported metastatic disease were on average 60.11 years old, 92.86% were female, and 7.14% male (14 vs. 1). Patients without metastases were on average 60.25 years old, 36.36% were male and 63.64% were female.

### Therapy

Data regarding performed therapy was available for 291 patients (47.55%). In 269 cases, surgery was performed, 22 patients did not receive surgery, and in 312 cases, no information about surgical procedures was available. None of the patients who did not receive surgery was reported dead during the observation period. Information on how the diagnosis of LAMN was made in these patients was not available.

Information about radiotherapy was available in 203 cases (33.19%) and one patient received radiation.

Information about performed chemotherapy was available for 212 patients (33.17%).

**Fig. 4:**
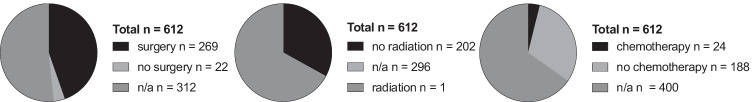
Performed therapy on LAMN cases, divided into surgical therapy, radiation, and chemotherapy; n/a, not available

A total of 88.68% (*n* = 188) did not receive chemotherapy and 11.32% (*n* = 24) did. In all patients that received chemotherapy, information about surgery was available and all but one underwent surgery. Eleven patients with T-stage T4 received surgery and chemotherapy; in 3 of these patients, an M-stage M1 was reported. For further clarification, see Fig. 4.

### Survival

**Fig. 5:**
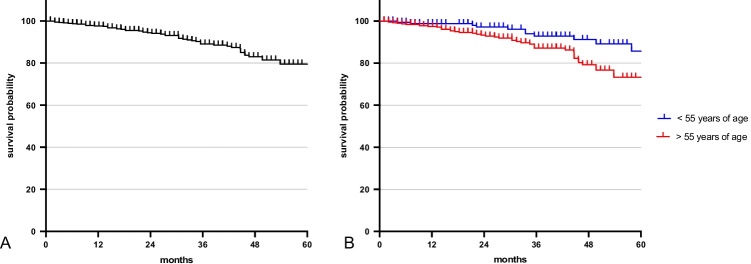
Survival probability: **A** overall survival; **B** survival probability below (blue) and above (red) the age of 55 years at time of diagnosis

Out of 612 patients, 67 passed away within the observation period. Overall 5-year survival rate was 79.52% (95% CI 73.12–84.57) (Fig. 5).

For the analysis, patients were divided into two groups: younger or older than the age of 55 at time of diagnosis. A total of 182 patients were younger than 55 years and 13 of them died within the observation period. The five-year survival for patients < 55 years of age was estimated at 85.77% (95% *CI* = 73.15–92.77). A total of 430 patients were older than 55 years and 48 of these patients died within the observation period. The five-year survival rate was 73.27% (95% CI 63.83–80.61). The log-rank-test revealed a significant lower survival probability for patients above 55 years of age compared to patients younger than 55 years (*P* = 0.0052).

The cause of death according to the ICD-10 classification system was described in 33 patients. Fourteen patients died of pneumonia, myocardial infarction, leukemia, hernia, sepsis, bowel obstruction, perforation, or ischemia. In 5 patients, the cause of death was marked unclear and further, 5 patients had a malignant disease of unknown origin. Malignant tumor of the coecum was described as cause of death in three patients. Twice a malignant tumor of the uterus and in one patient a tumor of the pancreas was named as cause of death. One patient died of a fracture and another one of an infection of the bowel.

Due to scarce data regarding tumor stage (available in only 15 patients), no further analysis was performed to evaluate the probability of death in relation to the tumor staging.

## Discussion

This is the first analysis on low-grade appendiceal neoplasms in Germany performed so far.

In 2018, the year with the highest number of reported LAMN cases, 108,247 appendectomies were performed [17, 18] and 142 cases of LAMN were reported to the ZfKD, leading to the presence of LAMN in 0.13% of all appendectomy specimen in 2018. The analysis by Smeenk et al. between 1995 and 2005 revealed an incidence of mucinous neoplasms of 0.52% in all appendectomy specimen in the Netherlands and described 876 cases. Average patient age at diagnosis was 61 years in female and 64 years in male patients. Females were predominantly affected (ratio m:w 1:1.4–1:1.8) [3]. Comparability to this analysis is limited due to a different classification of neoplasms. ICD-O-3 with the complementary code 8480/1 was only established in 2013.

In 2010, Lozano et al. described that 0.28% of appendectomy specimens contained LAMN in their collective [19]. The higher rates of LAMN in the above-mentioned studies might be a result of the incompleteness of reported LAMN cases to the ZfKD. Three federal states (Hamburg, Hesse, and Baden-Württemberg) did not report any cases during the observation period. Reasons for not reporting cases remain unknown, but it seems unlikely that no cases appeared in these states as they inhabit approximately 19 Mio. people, which is roughly 1/5 of the German population [20].

A rise in case numbers was visible from 2011 until 2015 with only a handful of cases in the first years of this observation period of this study. This observation might be due to the novelty of the LAMN diagnosis which was included into the “WHO classification of tumours” for the first time in 2010 [10] and into the ICD-O-3 in 2013 [16]. Until then, there was no consistent terminology of mucinous tumors of the appendix: mucocele, mucinous cystadenoma, borderline tumor, or mucinous tumor of uncertain malignant potential represents a selection of names that are no longer recommended to use [4]. With a uniform terminology, comparability is easier achieved, as well as entering patient-data in cancer registries.

Loftus et al. aimed to define predicting factors for appendiceal tumors and were able to identify some: advanced age, multiple comorbidities, atypical presentation of symptoms, and complicated appendicitis [1]. While we are not able to address the last 3 risk factors, advanced age can be confirmed as a risk factor with an average age of 62 years in our collective. In our population, women were predominantly affected (63.07% vs. 36.93% ratio: 1:0.585), which is in line with the results of Smeenk et al. and Loftus et al. even if it was not statistically significant in the study by Loftus et al. [1, 3].

Due to the rarity of LAMN, national or international standardized guidelines are not yet established. The current available literature suggests that appendectomy and in advanced stages peritonectomy, cytoreductive surgery, and HIPEC are recommended therapeutic strategies. While nodal status was available only in 56 patients, these patients were staged negative in all cases except one which is in line with existing studies. This emphasizes that oncological right hemicolectomy does not have any survival benefits as LAMN do not metastasize in lymph nodes [2, 9, 12]. Nevertheless, in studies, LAMN patients did receive extended surgery, in most cases right hemicolectomy [2, 21]. In light of the higher complication rate for colorectal resections, this should be avoided in LAMN patients [18, 22–25]. Referring to our own collective, in PMP cases in which right hemicolectomy was necessary to achieve complete cytoreduction, resected lymph nodes did not contain metastasis. This is supporting the recommendation that hemicolectomy should only be performed if necessary for complete cytoreduction [9]. Unfortunately, the provided data does not report the performed surgery in detail, therefore the data acquired for this manuscript cannot elucidate whether right hemicolectomy is beneficial.

Five-year survival of LAMN in this study was approximated at 79.52%, which is rather high compared to survival rates of colorectal cancer (approximately 62%) and can be compared to survival probability of breast cancer [13]. Patient age at time of diagnosis seems to be an important factor regarding survival favoring patients below the age of 55 (85.77% vs. 73.27%). Compared to colorectal or breast cancer, two of the most common tumor entities worldwide with standardized therapy and follow-up regimens, it can be assumed that LAMN patients frequently do not receive sufficient therapy or follow-up due to the rareness of these tumors which can affect the survival rates.

This manuscript does have some limitations: first and foremost, the information provided is incomplete regarding tumor stage and therapy. Even if data on therapy was available, this information does not include the type of surgery or drug and dosage of chemotherapy. Furthermore, it is not clarified if HIPEC is considered surgery or chemotherapy. Therefore, no statement or recommendation on the type of surgery or the use of chemo- or radiotherapy can be made with the acquired data.

Another interesting aspect is the fact that in 19 patients of the 152 (12.5%) in which t-stage was available, T1 and T2 were diagnosed. According to the current TNM-classification, T1 and T2 category is not existing for LAMN. Reasons why patients were diagnosed with these tumor categories are not available.

The provided data does not explain how the diagnosis was made in 22 patients who did not receive surgery. None of these patients was reported dead; therefore, diagnosis at the time of autopsy seems unlikely. The more likely reason seems to be misentry of the data.

## Conclusion

LAMN are a rare and unique tumor entity that is not yet fully investigated. This study shows the need for further investigation on LAMN as well as the need to establish guidelines for the treatment as most surgeons have already been or will be faced with the incidental finding of LAMN during surgery. By setting up a specialized registry, profound data can be acquired to provide further information on LAMN.
